# Altered serum interleukin-4 and monocyte chemoattractant protein-3 levels are associated with generalized anxiety disorder: a case-control study

**DOI:** 10.3389/ebm.2026.11144

**Published:** 2026-08-26

**Authors:** Most. Humayra Binta Rashid, Mehedi Islam, Sardar Mohammad Ashraful Islam, Md. Rabiul Islam

**Affiliations:** 1 Department of Pharmacy, University of Asia Pacific, Dhaka, Bangladesh; 2 School of Pharmacy, BRAC University, Dhaka, Bangladesh

**Keywords:** biomarker, cytokines, generalized anxiety disorder, IL-4, MCP-3

## Abstract

Generalized Anxiety Disorder (GAD) is a long-term mental health condition often associated with immune system dysregulation. While pro-inflammatory and anti-inflammatory cytokines are known to influence neuroinflammation, their specific roles in GAD remain less understood. This study investigates the relationship between serum levels of IL-4 and MCP-3 and GAD in a Bangladeshi population. This case-control study included 44 patients with GAD and 44 healthy controls (HCs). Participants were examined using the GAD-7 scale. Serum IL-4 and MCP-3 levels were measured using ELISA. Data were analyzed using t-tests, Spearman’s correlation, and Receiver Operating Characteristic (ROC) curve analysis to assess diagnostic performance. GAD patients had significantly lower serum IL-4 levels (11.82 ± 10.91 pg/mL) and higher MCP-3 levels (57.80 ± 19.85 pg/mL) compared to HCs (IL-4: 24.51 ± 15.36 pg/mL; MCP-3: 34.01 ± 15.75 pg/mL; p < 0.001). IL-4 levels showed a significant negative correlation with GAD-7 scores (r = −0.496, p < 0.001), while MCP-3 showed a positive correlation (r = 0.544, p < 0.001). ROC analysis indicated that MCP-3 had higher diagnostic accuracy (AUC = 0.848, sensitivity = 87.6%, specificity = 84.3%) compared to IL-4 (AUC = 0.782, sensitivity = 80.2%, specificity = 78.5%). Moreover, decreased IL-4 and elevated MCP-3 levels were found to be significantly associated with GAD severity, suggesting an immune imbalance. These cytokines may serve as promising diagnostic biomarkers and therapeutic targets for GAD. Further longitudinal studies are recommended to investigate this causal relationship and its underlying mechanisms.

## Impact statement

Generalized Anxiety Disorder (GAD) is a multifactorial psychiatric disorder characterized by dysregulation of the immune system. The specific role of inflammatory cytokines to the pathophysiology and development of GAD has not yet been fully elucidated. GAD patients showed altered serum IL-4 and MCP-3 levels compared to healthy subjects and the changes are associated with the severity scores of patients. The changes of serum IL-4 and MCP-3 levels indicated good diagnostic performances with high sensitivity and specificity. Therefore, the altered serum IL-4 and MCP-3 levels are associated with pathophysiology of GAD and these cytokines may serve as promising diagnostic biomarkers and therapeutic targets.

## Introduction

Generalized anxiety disorder (GAD) is a persistent neuropsychiatric condition that is characterized by intense and uncontrollable fear and worry for at least 6 months. It has become a significant public health concern as it impacts families, society, and the professional lives of those affected [1–3]. At present, the worldwide prevalence rate of GAD is between 3 and 6% and is increasing due to the complex lifestyles of modern civilization [4–6]. Without adequate intervention by national and international authorities, this condition can result in mental disability with reduced cognitive function and thus pose a considerable mental health concern, in addition to deteriorating the quality of life [2, 6]. Despite GAD’s high prevalence and negative socioeconomic impact, its pathophysiology and treatment strategies have yet to be characterized. Without having any precise knowledge of the pathogenesis of GAD, it has been found to be linked with neurochemical, neurobiological, genetic, psychological, environmental, and immunological factors [6]. Additionally, altered monoaminergic neurotransmissions, lowered GABA-mediated inhibitory transmission, or excessive activation of excitatory glutamatergic neurotransmission are found to be involved with the pathogenesis of GAD [7].

Several pharmacological treatments are currently in use for managing GAD. These include selective serotonin reuptake inhibitors (SSRIs), including fluoxetine, sertraline, escitalopram, paroxetine, and citalopram, and serotonin-norepinephrine reuptake inhibitors (SNRIs), including venlafaxine and duloxetine in addition to calcium channel blockers, pregabalin, and benzodiazepines; these medications cause common adverse effects, such as nausea, insomnia, sexual dysfunction, and weight changes, and may also lead to discontinuation symptoms [5, 6]. These therapeutic classes exert their activities by reversing altered monoaminergic transmission. In addition to pharmacotherapy, non-pharmacological interventions, including psychological therapies and stress management strategies, are widely recommended for the effective management of GAD symptoms [8]. Nevertheless, the effectiveness of these interventions has yet to be demonstrated, as half of the patients do not respond to these drugs. Moreover, the recurrence of the symptoms is seen in at least 30% of the patients [4, 8, 9]. Lower patient adherence and compliance of patients with these drugs have also been reported in studies: these are responsible for the discontinuation of these pharmacotherapeutics [6]. These findings underscore the need for further investigation into the underlying causes and mechanisms of the progression of GAD and the discovery of novel therapeutics. In addition, an operative and effective biomarker for the early detection of GAD could be successful in the treatment and management of the disease.

Since our current pharmacotherapeutic interventions, which are mainly oriented against the altered monoaminergic neurotransmitter system, are not achieving much success in terms of getting responses from affected patients, this suggests the involvement of other factors in the pathogenesis of GAD. A dysregulated immune system is becoming increasingly important in the pathogenesis and development of GAD, and several recent studies have also established an interrelation between a dysregulated immune system and the development of GAD [9–12]. Preclinical studies on animal models have also demonstrated the development of anxiety upon the introduction of pro-inflammatory cytokines such as L-1β, IL-1, IL-6, and TNF-α. These impacts were reversed or attenuated by the incorporation of anti-inflammatory cytokines such as IL-4, IL-10, or the introduction of antagonists for the respective cytokines [13–16]. These findings from preclinical studies can be further extrapolated to clinical studies, which have reported significantly higher levels of pro-inflammatory cytokines in GAD patients alongside lower anti-inflammatory cytokines when compared with healthy individuals [1, 17–23]. More studies have confirmed the significant association between the pro-inflammatory cytokines IL-6 and TNF-α with anxiety scores [24]. However, a few studies have also reported no meaningful alterations in anti-inflammatory or pro-inflammatory cytokine levels in GAD patients and, in some instances, with lowered levels of pro-inflammatory cytokines when compared with healthy volunteers [2, 25, 26]. These anomalies in the research findings further necessitate the investigation of these cytokines in the pathophysiology of GAD.

Microglia, the resident macrophages of the central nervous system (CNS), are the principal inflammatory cells, normally existing in a resting (surveillant) state [27]. They become activated under pathological conditions and accumulate at sites of injury or lesions. These activated microglia are of two subtypes: classically activated M1 phenotypes and alternatively activated M2 phenotypes [27]. The M1 phenotype is responsible for pro-inflammatory action by increasing the production of pro-inflammatory cytokines, for example, TNF-α and IL-6. On the contrary, the M2 phenotype is responsible for anti-inflammatory action due to the increased production of anti-inflammatory cytokines such as TGF-β and IL-10 [27, 28]. Furthermore, these anti-inflammatory cells can also suppress the response to pro-inflammatory cytokines [29] and thus contribute to neuroprotection. IL-4 has been reported by several studies to have neuroprotective potential by inciting alternatively activated M2 microglia or macrophages [30, 31]. Resolvin D1, a significant anti-inflammatory lipid mediator, has been found to accelerate the IL-4-induced STAT/PPARγ signaling pathway that alternatively activates microglia/macrophages [32]. Another study has reported that IL-4 has inhibitory properties on M1 activation and promotes M2 activation after intracerebral hemorrhage (ICH). Thus, IL-4 enhances the anti-inflammatory response [31]. Furthermore, administration of IL-4 in thrombin-induced BV2 cell and mouse ICH models may cause the switching of the M1/M2 phenotype in a JAK1/STAT6-dependent manner, reported by Yang et al. (2020) to have neuroprotective properties [33].

Chemotactic cytokines, or chemokines, induce an inflammatory cascade either by chemotaxis of specific cell types or by activating the target cell population. Monocyte chemotactic proteins (MCPs) are one of the major sub-families of CC chemokines, and the members of this subfamily share approximately 65% amino acid identity [34]. MCP-3, a member of the MCP subfamily, shares characteristics of MCP-1 and Regulated on Activation, Normal T-cell Expressed and MCP-3 shares structural and functional features with MCP-1 and RANTES and acts as a potent chemoattractant for basophils and eosinophils [35]. Although MCP-3 is structurally and functionally similar to MCP-1, it is involved in the chemotaxis of dendritic cells and eosinophils [36]. MCP-3 binds to the CCR-2 receptor (which comprises CC-CKR-2a and CCR-2 b), which is largely found on monocytes and activated lymphocytes [37]. It also acts as a ligand for the CCR-1 (CC-CKR-1) receptor, which is known to bind MIP-1α and RANTES [38]. Thus, it plays an important role in immune cell migration, especially during inflammation. Despite several studies confirming the association of immune dysregulation with the development of GAD, few studies have investigated the role of cytokines in GAD. Therefore, the current article aimed to investigate the role of IL-4 and MCP-3 in the pathophysiology and progression of GAD.

## Materials and methods

### Study population

For this case-control study, data and blood samples were collected from 88 participants. The participants included 44 GAD patients and 44 healthy controls (HCs) who were matched based on their age and sex. Patients with GAD were recruited from the Department of Psychiatry, Bangladesh Medical University Hospital, Dhaka, and the HCs were recruited in Dhaka and its surrounding areas. A professional psychiatrist was employed to evaluate the participants based on DSM-5 criteria. The 7-item Generalized Anxiety Disorder (GAD-7) scale was utilized to assess the severity of anxiety symptoms [39]. Total scores were obtained in a range of 0–21 and were classified into 4 categories to define anxiety severity (scores of 0–4 indicate minimal anxiety, 5–9 mild, 10–14 moderate, and 15–21 severe). A questionnaire was designed prior to the study to record the sociodemographic profile of each participant. The purpose of the study was clearly explained to all participants, and written informed consent was obtained from each of them. This study followed the ethical guidelines of the Declaration of Helsinki, prioritizing the safety and rights of all participants.

Exclusion criteria for the study were as follows: comorbidity with other psychiatric disorders, such as MDD, PTSD, panic disorder, bipolar disorder, schizophrenia, social phobia, or any other mental illness; suffering from chronic hepatic or renal disorders, acute or chronic infectious diseases, malignancies or autoimmune diseases; cognitive or functional impairment preventing questionnaire completion; taking anxiolytics or antidepressant medications within 2 weeks of the study, and pregnancy. Individuals with any of the above were excluded from the study population as these conditions might have impacted blood cytokine levels.

### Blood sampling and serum isolation

Approximately 5 mL of blood was collected from the cephalic vein of each participant. The blood was allowed to clot by resting at room temperature for 1 h. Following clotting, the samples were centrifuged at 3,000 rpm for 15 min to separate the serum. The serum was then carefully transferred to Eppendorf tubes and stored at −80 °C until further analysis.

### Quantification of serum cytokine levels

IL-4 and MCP-3 were utilized as biomarkers in this study and estimated in the blood serum by the ELISA method (Boster Bio, USA). The protocol provided by the manufacturers was followed for the ELISA assays. For each well of the pre-coated 96-well microplate, 100 μL of the standard cytokine solution, samples, and controls were added. The plate was then covered and kept at 37 °C for 90 min. Once the incubation was complete, the seal was taken off, and the liquid inside the wells was discarded. Then, 100 μL of biotinylated anti-IL-4 or anti-MCP-3 antibody was added to each well, and the plate was incubated at 37 °C for 1 h. After the incubation period, the liquid from each well was carefully removed, and the wells were washed three times using 300 μL of wash buffer. In total, 100 μL of the avidin-biotin-peroxidase complex was added to each well, and the plate was incubated again at 37 °C for another 30 min. The liquid was removed once more, and the plate was washed five times using 300 μL of wash buffer each time. Then, 90 μL of the color-developing reagent (TMB) was added to every well, and the plate was left in a dark place at room temperature for 30 min. To bring the reaction to an end, 90 μL of stop solution was added to each well, and the absorbance was measured at 450 nm for all the samples. Cytokine levels were then calculated utilizing a standard curve and represented as pg/mL.

### Data interpretation and statistical analysis

For the analysis of the data, we used GraphPad Prism (Version 8.0.1) and SPSS (Version 25.0). Descriptive statistics were used to explore and summarize the differences in sociodemographic and clinical profiles across the groups. To determine if there were significant differences between patients and controls, a t-test was performed on continuous data, while categorical data were analyzed using a chi-square test. Error bar graphs were used to compare cytokine levels between the two groups. We performed a correlation analysis to see how different demographic and clinical variables were connected in these patients. Moreover, we utilized receiver operating characteristic (ROC) analysis to evaluate the ability of serum IL-4 and MCP-3 levels to distinguish GAD patients from healthy individuals. All the findings with a p-value under 0.05 were regarded as statistically significant.

## Results

### Sociodemographic profile of the study participants

Table 1 shows the socio-demographic characteristics of the GAD patients and HCs. There were no meaningful differences between them in terms of age, sex, marital status, education level, occupation, residence area, and smoking history (p > 0.05). Nonetheless, significant variation was observed in economic status and family history between the GAD patients and HCs with a p-value of less than 0.05.

**TABLE 1:** Sociodemographic characteristics of the study population.

Parameters	GAD patients		Healthy controls		p value
	Frequency (n)	Percentage (%)	Frequency (n)	Percentage (%)	
Age in years	​	​	​	​	0.673
18–25	14	31.82	16	36.36	​
26–35	20	45.46	15	34.10	​
36–45	5	11.36	8	18.18	​
46–60	5	11.36	5	11.36	​
Sex	​	​	​	​	0.520
Male	26	59.09	23	52.27	​
Female	18	40.91	21	47.73	​
BMI (kg/m^2^)	​	​	​	​	0.340
Below 18.5 (CED)	6	13.64	3	6.82	​
18.5–25.0 (normal weight)	29	65.91	27	61.36	​
Above 25.0 (overweight or obesity)	9	20.45	14	31.82	​
Marital status	​	​	​	​	0.133
Married	21	47.73	28	63.64	​
Unmarried	23	52.27	16	36.36	​
Education level	​	​	​	​	0.209
Illiterate	3	6.82	3	6.82	​
Primary	3	6.82	0	0.00	​
Secondary	16	36.36	12	27.27	​
Graduate and above	22	50.00	29	65.91	​
Occupation	​	​	​	​	0.201
Business	2	4.55	2	4.55	​
Service	11	25.00	12	27.27	​
Housewife	10	22.73	9	20.45	​
Student	14	31.82	14	31.82	​
Unemployed	7	15.90	7	15.91	​
Economic status	​	​	​	​	**0.019**
Low	8	18.18	15	34.09	​
Medium	22	50.00	25	56.82	​
High	14	31.82	4	9.09	​
Residence area	​	​	​	​	0.346
Rural	26	59.09	32	72.73	​
Urban	18	40.91	12	27.27	​
Smoking history	​	​	​	​	0.398
Non-smoker	42	95.45	40	90.91	​
Smoker	2	4.55	4	9.09	​
Family history of GAD	​	​	​	​	**0.002**
Yes	0	0.00	9	20.45	​
No	44	100.00	35	79.55	​

Abbreviations: GAD, generalized anxiety disorder; BMI, body mass index; CED, chronic energy deficiency. Significant *p* values are indicated as bold.

### Clinical profiles and laboratory findings of the study population

**FIGURE 1:**
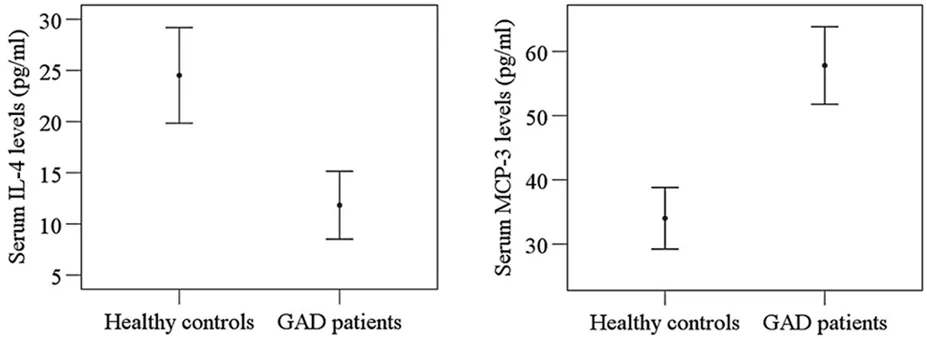
Error bar graphs showing the comparison of serum IL-4 and MCP-3 levels between GAD patients and healthy controls. Values are expressed as the mean ± standard error of the mean (SEM). Error bars indicate SEM. Statistical comparisons were performed using an independent samples t-test. P < 0.05 indicates statistical significance.

The clinical profiles and the laboratory findings of the study population are presented in Table 2. Although no meaningful variation was noted between GAD patients and HCs in terms of age (p = 0.682) and BMI (p = 0.524), significant differences were observed between them in GAD-7 scores and serum levels of IL-4 and MCP-3. GAD-7 scores were found significantly higher in GAD patients (13.41 ± 3.24) compared to healthy volunteers (4.56 ± 2.21). Conversely, significantly reduced levels of serum IL-4 were found in GAD patients (11.82 ± 10.91) when compared with HCs (24.51 ± 15.36). Additionally, significantly higher levels of serum MCP-3 were observed in GAD patients (57.80 ± 19.85) compared to HCs (34.01 ± 15.75) (Table 2; Figure 1).

**TABLE 2:** Clinical features and laboratory findings of the study participants.

Parameters	GAD patients Mean ± SD	Healthy controls Mean ± SD	p value
Age (in years)	30.91 ± 10.55	31.82 ± 10.21	0.682
BMI (kg/m^2^)	22.28 ± 3.26	23.46 ± 3.47	0.524
GAD-7 scores	13.41 ± 3.24	4.56 ± 2.21	<0.001
Serum IL-4 levels (pg/mL)	11.82 ± 10.91	24.51 ± 15.36	<0.001
Serum MCP-3 levels (pg/mL)	57.80 ± 19.85	34.01 ± 15.75	<0.001

Abbreviations: BMI, body mass index; GAD, generalized anxiety disorder; IL-4, interleukin-4; MCP-3, monocyte chemoattractant protein-3; SD, standard deviation; GAD-7, 7-item generalized anxiety disorder scale.

### Association between IL-4 and MCP-3 with GAD severity

A Spearman’s correlation study was utilized to examine the interrelation between IL-4 and MCP-3 with the severity of GAD. A statistically significant negative association was noted between age and IL-4 (p = 0.042), IL-4 and GAD-7 score (p < 0.001), and IL-4 and MCP-3 (p = 0.027). A statistically significant positive association was observed between MCP-3 and the GAD-7 score (p < 0.001). Age of GAD patients is not associated with GAD-7 score and MCP-3 levels according to the present study. Furthermore, correlation analysis showed that BMI was not associated with IL-4, MCP-3, or GAD-7 scores (Table 3).

**TABLE 3:** Spearman’s correlation study on various research parameters among GAD patients.

*Correlation parameters*	*r*	*p*
Age and GAD-7 score	0.024	0.827
Age and IL-4	−0.217	**0.042**
Age and MCP-3	−0.096	0.371
BMI and GAD-7 score	−0.174	0.104
BMI and IL-4	0.031	0.775
BMI and MCP-3	−0.075	0.489
IL-4 and GAD-7 score	−0.496	**<0.001**
MCP-3 and GAD-7 score	0.544	**<0.001**
IL-4 and MCP-3	−0.236	**0.027**

Abbreviations: BMI, body mass index; GAD, generalized anxiety disorder; IL-4, interleukin-4; MCP-3, monocyte chemoattractant protein-3; GAD-7, 7-item generalized anxiety disorder scale. Significant *p* values are indicated as bold.

### Assessment of the diagnostic efficacy of serum IL-4 and MCP-3 levels by receiver operating characteristic curve analysis

**FIGURE 2:**
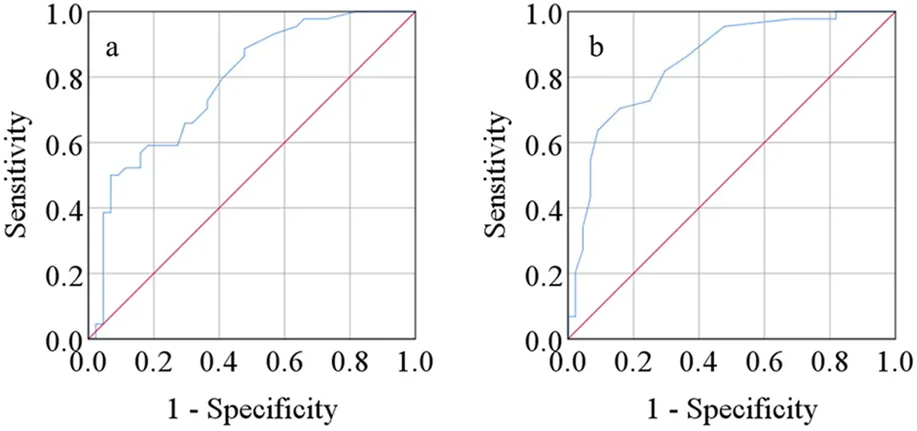
Receiver operating characteristic (ROC) curve analysis. **(a)** ROC curve analysis of serum IL-4 levels among study participants (p < 0.001) with area under the curve (AUC), sensitivity, and specificity of 0.782 (95% CI: 0.686–0.878), 80.2%, and 78.5% at a cutoff value of 15.0 pg/mL. **(b)** ROC curve analysis of serum MCP-3 levels among study participants (p < 0.001) with area under the curve (AUC), sensitivity, and specificity as 0.848 (95% CI: 0.767–0.930), 87.6%, and 84.3% at a cutoff value of 45.0 pg/mL.

To assess the diagnostic potential in distinguishing GAD patients from HCs, a receiver operating characteristic (ROC) analysis was performed to evaluate the ability to distinguish GAD patients from healthy controls (HCs) based on reduced serum IL-4 levels and elevated serum MCP-3 levels (Figure 2). Analyzing the ROC curve of serum IL-4 revealed a prognostic result with an area under the curve (AUC) value of 0.782 (95% CI: 0.686–0.878) and sensitivities and specificities of 80.2% and 78.5% at a cutoff value of 15.0 pg/mL. In comparison to IL-4, serum MCP-3 level appeared with greater efficacy in distinguishing GAD patients from HCs, with AUC, sensitivity, and specificity as 0.848 (95% CI: 0.767–0.930), 87.6%, and 84.3% at cutoff value 45.0 pg/mL.

## Discussion

Anxiety develops from a complicated mix of our neurological function, genetic inheritance, and environmental variables [40]. One key aspect of developing GAD is the dysregulation of the immune system, although its contribution is often overlooked [10]. Patients with GAD in this Bangladeshi group had elevated levels of the pro-inflammatory cytokine IL-2 and reduced levels of the anti-inflammatory cytokine IL-10 when compared to HCs. This imbalance suggests that patients with GAD may be experiencing increased inflammation because of their body’s inability to regulate inflammatory and anti-inflammatory responses [7]. Therefore, this investigation was designed to assess the relationship between the disease mechanisms of GAD and the anti-inflammatory cytokine IL-4 and the pro-inflammatory cytokine MCP-3.

IL-4 levels were found to be significantly lower in GAD patients. This illustrates the association of decreased anti-inflammatory cytokines in the progression of GAD. IL-4, an important anti-inflammatory cytokine produced by Th2 cells, helps balance the immune system by counteracting pro-inflammatory responses driven by Th1 and Th17 cells [41, 42]. IL-4 reduces the production of several inflammatory molecules, including cytokines such as IL-1, IL-6, and TNF-α, and chemokines such as IL-8 and MCP-3 [41, 43]. It also suppresses harmful substances, such as prostaglandins and reactive oxygen species [44]. Additionally, IL-4 also enhances the expression of natural anti-inflammatory molecules such as the IL-1 receptor antagonist (IL-1Ra) and the IL-1 receptor type II (IL-1RII), which help control inflammation in the body [45]. Its protective role has also been shown in conditions such as psoriasis, type 1 diabetes, arthritis, and multiple sclerosis [46]. Therefore, a reduction in the anti-inflammatory cytokine IL-4 may lead to an overexpression of pro-inflammatory cytokines, potentially playing a role in the progression of GAD. Our findings are further strengthened by the significant but negative association between IL-4 and GAD-7 score, implying a potential link between lower serum IL-4 levels and the development of GAD and GAD-7 score and MCP-3. However, our findings do not align with those of a number of previous studies, which have reported either no meaningful differences in IL-4 levels [47] or a significant increase in these levels in patients in comparison to controls [48, 49].

Conversely, pro-inflammatory cytokine MCP-3 levels were found to be significantly elevated in GAD patients, with a significant positive association with GAD-7 scores. MCP-3 shares structural and functional similarities with MCP-1 and acts as a strong chemoattractant for monocytes, T cells, and NK cells [50]. Studies using MCP-3 knockout (MCP-3^−/−^) mice have demonstrated that MCP-3 functions as a key CCR2 ligand, facilitating the recruitment of monocytes to the inflamed tissues [51]. Through this mechanism, MCP-3 plays an important role in directing immune cells to sites of inflammation, contributing to the initiation and maintenance of inflammatory responses, which may potentially be linked to the development and severity of GAD [51]. Our study further supports the cytokine hypothesis of anxiety disorders, which claims that pro-inflammatory cytokines contribute to anxiety symptoms by reducing serotonin production or increasing its reuptake, thereby altering the serotonergic system in the CNS [52]. The positive association with the GAD-7 score also signifies the potential development of GAD with increased levels of this pro-inflammatory cytokine, MCP-3.

The ROC analysis highlighted the potential of these biomarkers to differentiate between healthy individuals and those with anxiety disorders, suggesting their usefulness in both diagnosis and monitoring disease progression. Including IL-4 and MCP-3 in diagnostic approaches could improve accuracy and support the early identification of individuals at risk for GAD, thereby allowing for timely intervention. The role of inflammatory biomarkers in diagnosing mental health conditions has been gaining support in recent research. For instance, serum levels of IL-6, a pro-inflammatory cytokine, have been suggested as a way to monitor how GAD patients respond to SSRI treatment [53]. However, in our study, MCP-3 appeared to be a more promising biomarker than IL-4, showing higher sensitivity and specificity. The ROC analysis revealed area under the curve (AUC) of 0.848, with 87.6% sensitivity and 84.3% specificity which indicates that MCP-3 has strong potential to distinguish GAD patients from HCs. Nevertheless, further longitudinal studies are needed to confirm how IL-4 and MCP-3 are connected to disease severity and the underlying mechanisms of GAD.

Another potential implication of our findings is the possibility that the IL-4 and MCP-3 immune pathways could be explored as a target for developing new anxiolytic, antidepressant, or anti-inflammatory drugs. These treatments could work by targeting the soluble IL-4 or MCP-3 proteins themselves or by interfering with their receptor-mediated signaling pathways [54]. Currently, several anti-inflammatory monoclonal antibodies are being developed to target specific pro-inflammatory cytokines. For instance, secukinumab, ixekizumab, and bimekizumab are designed to block IL-17A, while ustekinumab targets IL-23A. These therapies are used to help manage a range of autoimmune conditions, including inflammatory bowel disease (IBD), ulcerative colitis, psoriatic arthritis, and rheumatoid arthritis [55, 56]. Our findings suggest that the IL-4 and MCP-3 immune axis may be linked to the severity of anxiety, either by actively contributing to the development of GAD through increased neuroinflammation in brain areas related to fear and anxiety, or by reflecting the biological processes involved in the disorder. In either case, these cytokines appear to play a role in the disease process and could be promising targets for developing new anxiolytic or anti-inflammatory treatments. However, the immune system is complex and finely balanced, so creating targeted treatments for GAD depends on a deep understanding of the specific immune processes that contribute to the condition.

The strengths of our study are that we used multiple analyses to carefully examine how serum levels of IL-4 and MCP-3 relate to anxiety severity, and whether these cytokines could help predict the risk of developing GAD. We set clear inclusion and exclusion criteria for participant selection to ensure a more uniform group. This careful design greatly helped us reduce the influence of other factors such as age, sex, BMI, other health conditions, and medications, which could all have affected cytokine levels.

While our study provides important insights, it does have some limitations that we should acknowledge. Although we observed a link between IL-4 and MCP-3 levels and anxiety severity, how this immune axis contributes to GAD symptoms remains unclear. Our relatively small sample size may not fully represent the broader Bangladeshi population and could affect the robustness of the findings. We also could not account for all possible confounding factors, such as genetic variations, lifestyle habits, or environmental exposures, which may influence cytokine levels. Also, since our study was case-control in nature, it can only show associations, not causal relationships. Another limitation of the present study is the lack of detailed information regarding psychopharmacological treatment, including specific medications prescribed, dosages, and therapy duration. As antidepressants and other psychotropic medications may modulate inflammatory pathways and cytokine expression, the absence of these data may have confounded the observed associations between serum inflammatory biomarkers and GAD. Consequently, the findings should be interpreted with caution. Future prospective longitudinal studies incorporating comprehensive medication histories and clinical course variables are needed to clarify the temporal dynamics of immune dysregulation throughout the progression of GAD.

## Conclusion

Our study highlights the significant interrelation between altered levels of IL-4 and MCP-3 with the pathophysiology of GAD. The decreased anti-inflammatory IL-4 and increased pro-inflammatory MCP-3 suggest an imbalance in immune regulation that may contribute to GAD pathophysiology. These findings support the growing body of evidence indicating that immune dysregulation plays a role in anxiety disorders. Targeting the IL-4/MCP-3 axis could open new avenues for developing anxiolytic or anti-inflammatory therapies. Further longitudinal research is needed to better understand the immune mechanisms involved in GAD and to validate these biomarkers for clinical use.

## Data Availability

The original contributions presented in the study are included in the article/supplementary material, further inquiries can be directed to the corresponding author.
